# Other ways of communicating the pandemic - memes and stickers against COVID-19: a systematic review

**DOI:** 10.12688/f1000research.51541.1

**Published:** 2021-04-13

**Authors:** Jeel Moya-Salazar, Betsy Cañari, Lucía Gomez-Saenz, Hans Contreras-Pulache

**Affiliations:** 1 Department of Pathology, Hospital Nacional Docente Madre Niño San Bartolomé, Lima, Lima, 51, Peru; 2 Faculties of Health Science, Universidad Norbert Wiener, Lima, Lima, 51, Peru

**Keywords:** Smartphone, COVID-19, eHealth Strategies, Cell Phone Use, SARS-CoV-2, Health Promotion

## Abstract

**Background: **In the midst of the coronavirus disease 2019 (COVID-19) pandemic, there are many ways to communicate hygiene measures, such as memes and stickers that are widely used on social networks. We carried out a systematic review in order to determine the impact of stickers and memes as tools to face the COVID-19 pandemic, following the PRISMA guide.

**Methods:** The search was carried out in scientific databases (MEDLINE / PubMed, ScientiDirect, Scielo, LILACS, and Latindex), and in public pre-publication servers (bioRxiv, SocArXiv, medRxiv and Preprints). The publications were identified using the terms (((meme) OR (sticker)) AND ((COVID-19) OR (SARS-COV-2)) AND (WhatsApp)) and the corresponding translations for Spanish and Portuguese.

**Results: **In the initial search, 8434 studies were obtained, 7749 in Preprints, 446 in SocArXiv, 145 in ScientDirect, 82 in medRxiv, and 12 in PubMed. No studies were found in LILACS, Latindex, Scielo, or bioRxiv. Of the 51 studies included as eligible, all were eliminated for not meeting the study inclusion criteria. The majority (40 studies) were eliminated as studies were publications related to the social aspects related to COVID-19, but did not develop an analysis of stickers or memes.

**Conclusions: **No studies were identified that met the inclusion criteria related to the role of stickers and memes as tools to face the COVID-19 pandemic. More studies are needed to estimate its role as a means of communication in health.

## Introduction

In the entire history of humanity, around 20 pandemics have been posed a danger to global health and have shaken the health systems of each era. The recent coronavirus disease 2019 (COVID-19) pandemic, caused by the new type of coronavirus (SARS-CoV-2), is causing high rates of infections and mortality in almost all countries in the world. ^1^ The difference between COVID-19 which is ~12 months-old, and its equivalents such as the Spanish flu (1918-1919), the Black Death (1347-1321), or smallpox (1520), is that COVID-19 develops in the middle of the technological era where natives and digital immigrants coexist in an environment of fundamental interconnection.

In the midst of a videocracy, governments worldwide have developed primary and secondary prevention mechanisms against this pandemic. The main tools in almost all affected countries, in the absence of vaccines or standardized treatments, have been quarantine (social isolation) and the use of optimal hygiene measures (through hand washing and social distancing). ^2^ To understand both prevention strategies, it is necessary for communities to integrate these “new” concepts as informational and educational tools, and apply them to their daily lives.

The media undertake this purpose by providing massive amounts of information about COVID-19, monopolizing all broadcast schedules. However, language as a primary tool for this purpose should not only be represented by the use of textual-written language. The brain, being a semiotic system, has the ability to read various forms of language (mathematical formulas, icons, cinema, figures, etc.), and in fact it does, more so now that people are influenced by the stimuli that the *image* offers. It is understood that we read more than language that only depends on letters and words. ^3^ For this reason, communications increasingly hover over “new” forms of language such as the use of symbols and figures (emojis). Its benefits and challenges have even been considered in medical communications. ^4^

**Figure 1.**
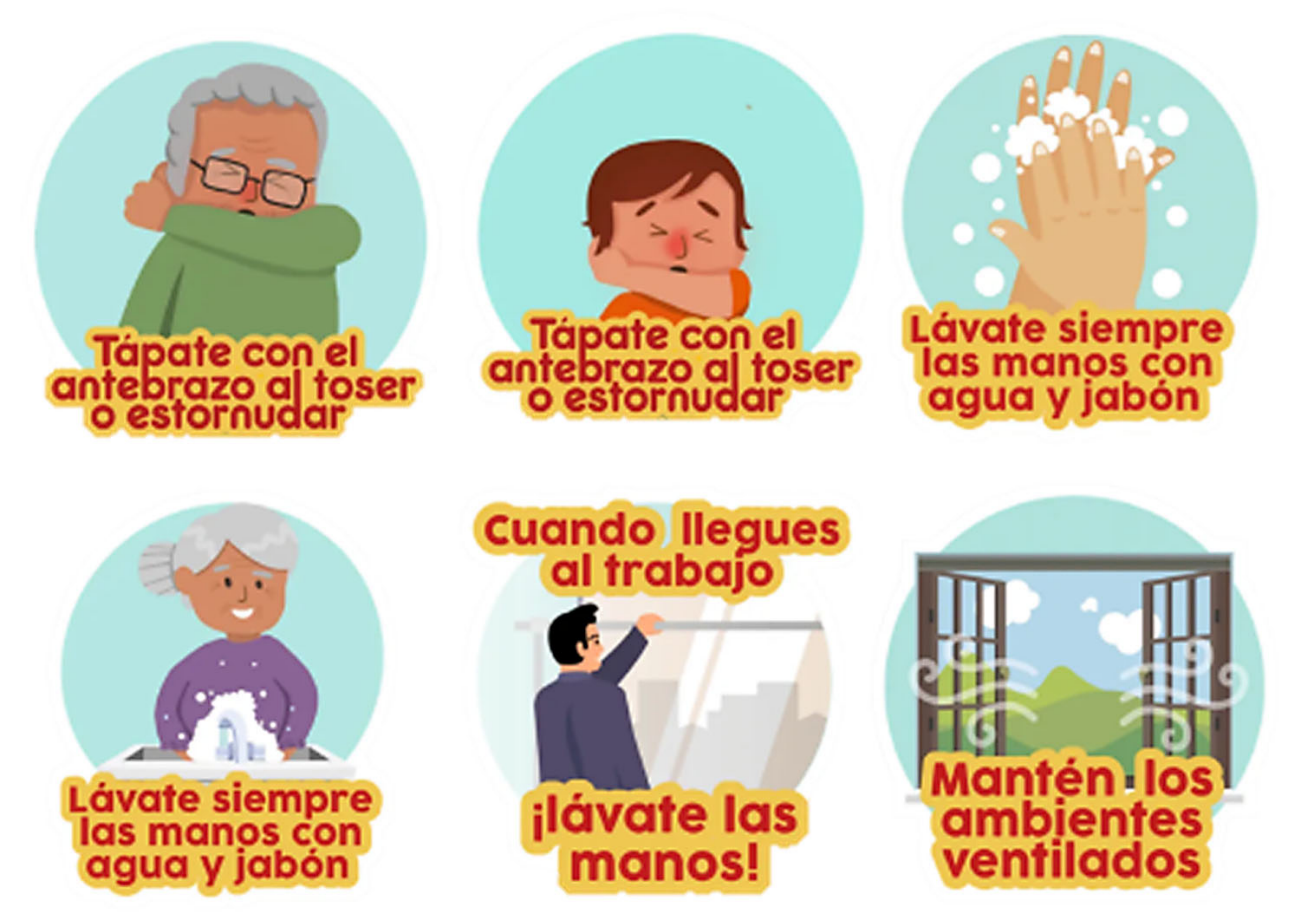
“Independent” stickers used in WhatsApp as information tools on hand washing. [Has been reproduced respecting the intellectual property rights of WhatsApp sticker users].

Apparently, these images are not limited to the definitions established by language, and on the contrary they generate a more expressive and cosmopolitan language. ^5^ Like emojis, stickers also seem to represent a key piece of contemporary communication, including in providing information on prevention measures (such as hand washing) during the COVID-19 pandemic ( Figure 1).

**Figure 2.**
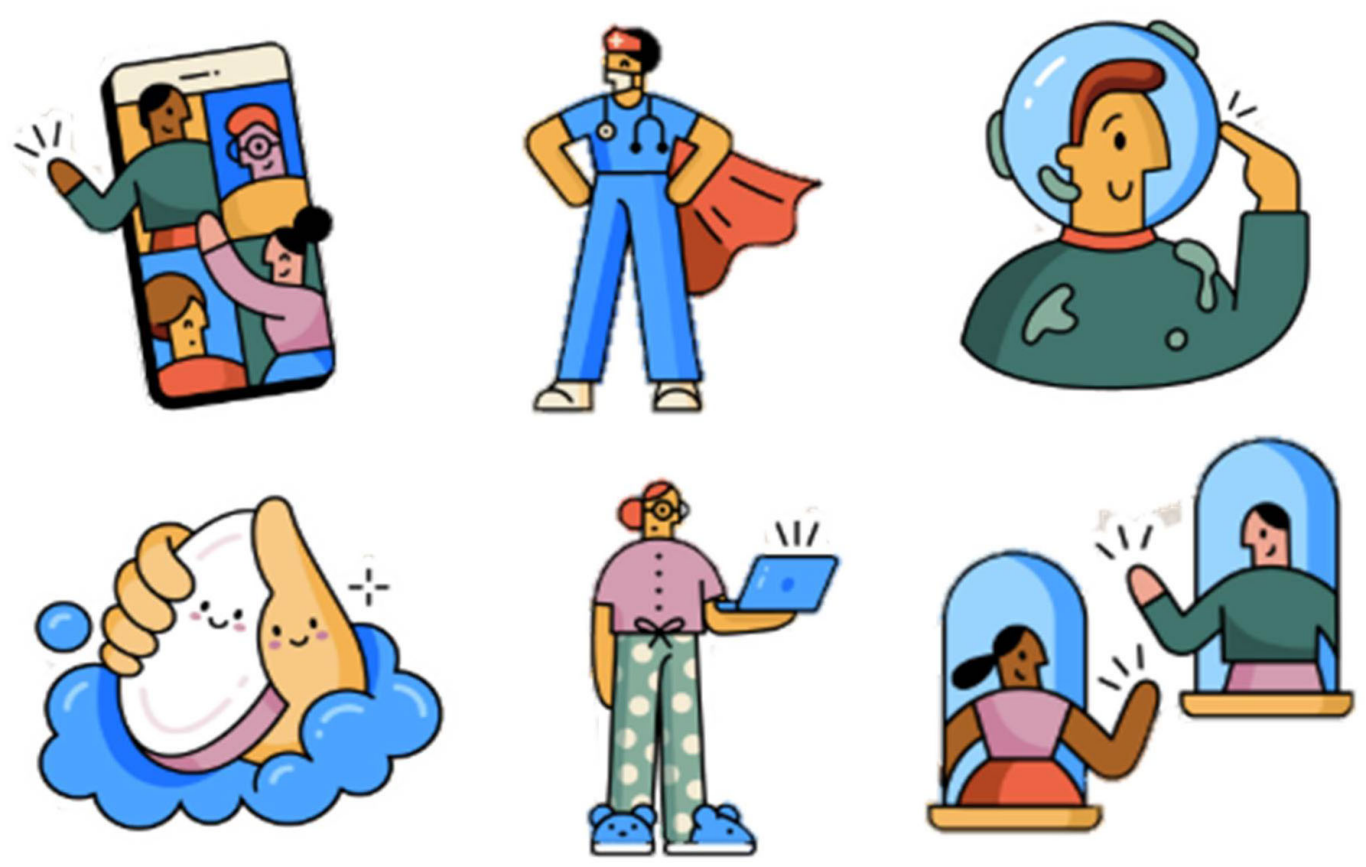
Together at home: WhatsApp stickers co created with the World Health Organization in the face of the coronavirus disease 2019 (COVID-19) pandemic. [Has been reproduced respecting the intellectual property rights of the World Health Organization’ WhatsApp stickers].

As epidemic multimodal characters, ^6^ stickers have been considered by the World Health Organization (WHO) as an important tool to face the pandemic, detailing basic prevention measures and changes in social behavior, such as new citizen behaviors of distancing ( Figure 2). In collaboration with WhatsApp, the WHO developed a set of stickers in April 2020 called “Together at home” in 10 languages, seeking to highlight the work of health professionals, prevention measures (such as hand washing), and activities promoted during confinement.

Although stickers have a growing impact as tools for the dissemination of prevention measures against COVID-19, memes have not yet been considered as tools for the same purpose, even though memes are massively used one-person productions that reflect the current moment with ironic interpretations.

In this study, we conducted a systematic review to determine the impact of stickers and memes as tools to face the COVID-19 pandemic.

## Methods

### Study design and eligibility criteria

In order to assess the role of stickers and memes in the face of the current pandemic, we developed a systematic review following the recommendations of the PRISMA guide (Preferred Reporting Items for Systematic Reviews and Meta-Analyzes). ^7^^,^^23^ We consider eligible publications that met the following inclusion criteria: (i) original studies (prospective or retrospective), trials, narrative reviews, case-control studies, perspectives, and scientific letters, and (ii) articles in English, Portuguese, and Spanish. We consider as exclusion criteria articles of reflection, systematic reviews, meta-analysis, and editorial letters (correspondence). This systematic review was registered in PROSPERO (CRD42020210205, date: 12/16/2020), complying with international requirements in good research practices and publications of systematic reviews.

### Search strategy

We carried out a systematic search in the main scientific search engines (PubMed, Scopus, Scielo, ScientDirect, LILACS, and Latindex), in public pre-publication servers (bioRxiv, SocArXiv, medRxiv and Preprints) and in Google Scholar and Yahoo! Pre-publication search engines were used since the global emergency context led to the promptness of publication with the extensive use of these search engines.

**Figure 3.**
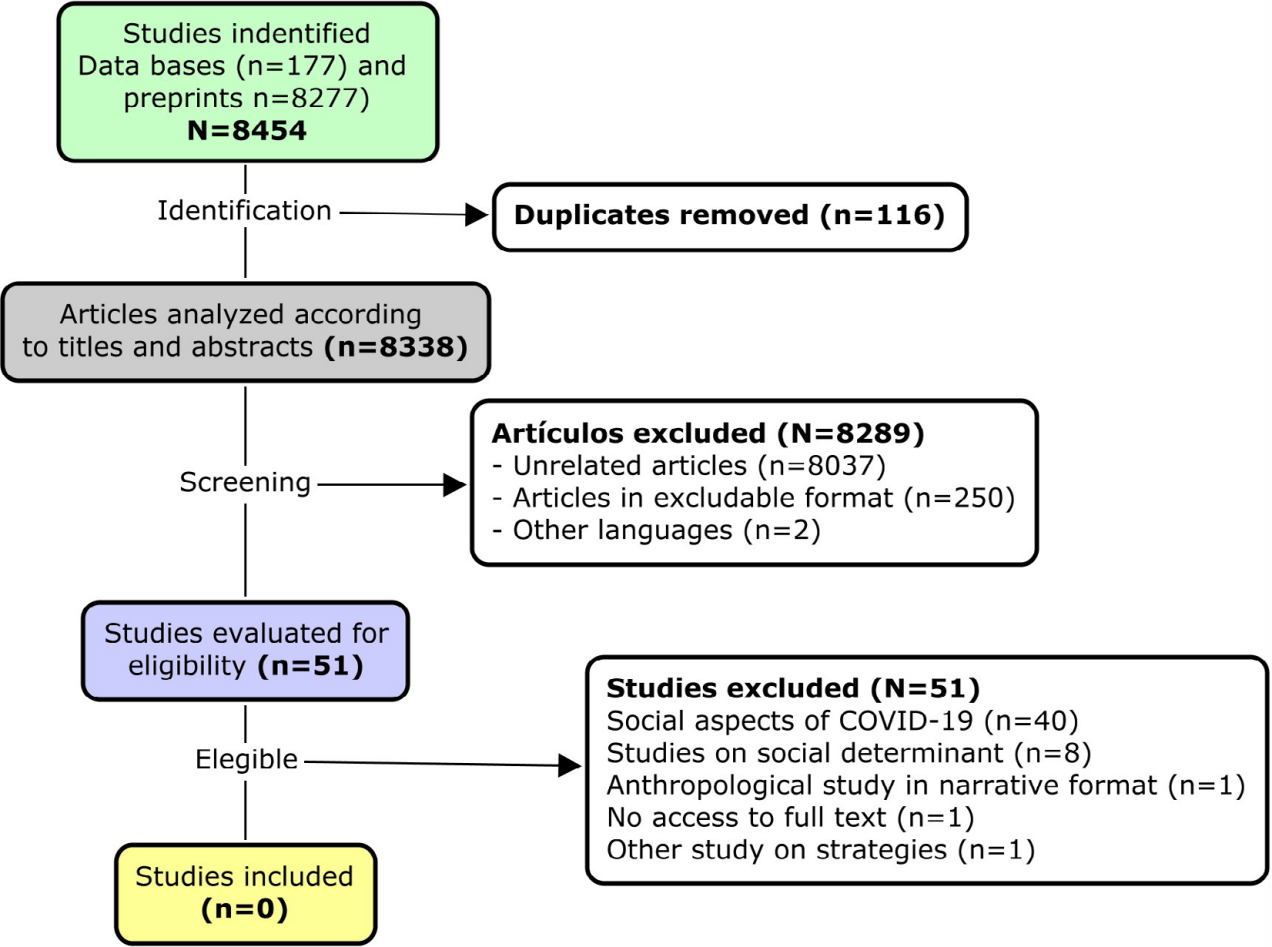
PRISMA flow chart for the selection of studies on stickers and memes against COVID-19.

The publications were identified using the terms (((meme) OR (sticker)) AND ((COVID-19) OR (SARS-COV-2)) AND (WhatsApp)) and the corresponding translations for Spanish and Portuguese [(((meme) OR (sticker) OR (stickers)) AND ((COVID-19) OR (SARS-COV-2)) AND (WhatsApp))]. The manual search was carried out between December 22 and 30, 2020 and the review was carried out independently by two authors to verify the inclusion criteria of the studies according to PRISMA ( Figure 3).

We consider as standard studies those that detailed the impact of memes and/or stickers on the promotion and prevention of COVID-19, those that described the types of memes and stickers used in social networks, and the organizations that promote their use.

### Data extraction and analysis

Data extraction was performed by two investigators (J.M-S and H.C-P.) according to the predefined protocol. The studies were included in a data matrix (in IBM SPSS v21.0, Armonk, USA) according to the objectives of the study, this base was checked twice by the researchers. A formal descriptive analysis of the studies found was carried out.

## Results

In the study selection, the initial search obtained 8454 studies, consisting of 8277 in Preprints, 446 in SocArXiv, 145 in ScientDirect, 82 in medRxiv, 20 in Scopus and 12 in PubMed. No studies were found in LILACS, Latindex, Scielo, or bioRxiv. Of the 51 studies included as eligible, all were eliminated for not meeting the study inclusion criteria. The majority (40 studies) were eliminated as studies were publications related to the social aspects related to COVID-19, but did not develop an analysis of stickers or memes. Other publications included a newspaper account about quarantine ^8^ and the use of WhatsApp in times of pandemic (but without relevance for the use of memes and stickers). ^9^^,^^10^

During the study period, no studies were identified that meet the standard required to analyze the impact of stickers and memes as communication tools to face the COVID-19 pandemic, therefore there is insufficient firm evidence that can explain their impact on programs of prevention, given the null evidence found, despite the fact that many governments and international institutions are using these communication elements as information strategies in the face of the current pandemic.

## Discussion

This systematic review showed that there is no evidence available on the role of stickers and memes as tools to face the COVID-19, despite the fact that many governments and international institutions are using these message elements as communication strategies against the current pandemic.

By not obtaining eligible studies, this study is classified as an “empty review”. ^11^ The empty reviews are also tools to improve existing evidence. Like scoping reviews, ^12^ these reviews are particularly important since they show the null existence of studies on the area of interest (the role of memes and stickers against COVID-19). We well know that current health strategies are using all the available technological resources to face the inclemency of the pandemic, however, there are few reports on the role of digital media actors today. ^13^ The limitation of this study was that thesis or in-house institutional publications were not included since we focus on the searching the main databases.

Since digital communication began several decades ago, mechanisms have been established to provide rapid, massive, secure, and free access to communication using the benefits of the internet. With the explosion in popularity of digital telephones and the growth of applications (apps), the world is becoming interconnected within the imperative need for human communication.

WhatsApp (Facebook, MA, US) allows free instant mobile messaging through texts, phone calls, videos, and also allows sharing multimedia files individually, or in both national and international groups. ^5^ More than a billion people currently use this application in many fields, including in the university setting. ^14^^,^^15^ This system has an easy-to-use platform that shares the same mobile contacts, allows communication in different formats (text, voice messages, images, emojis, and recently stickers and moving stickers (gif)) clearly dominating instant messaging worldwide.

Stickers (artistic adhesives), which were initially introduced in digital communication by Line (LINE corp., Tokyo, Japan) with payments for access to these, and then Telegram (Telegram FZ-LLC, London, UK) with a more open sticker platform, so that the user can design their own stickers and can share them. This influenced WhatsApp’s adoption of stickers, since 2018, as a language for all its users, whether they are millennials, centenials, etc., enabling creativity in their design that surpasses the Unicode of emojis.

Stickers as a powerful communication tool (where users can create, modify and share at their convenience) and the number of WhatsApp users provide an inclusive and massive communication environment of the personal and the social. ^16^ The stickers are not limited to the definitions that language establishes, and on the contrary they generate a more expressive and cosmopolitan language. Stickers have a gradually relevant role in digital communication, replacing other forms of language. ^17^

Just as emojis ^4^^,^^18^ can offer new advantages in health communication, stickers seem to represent a key piece of contemporary communication, even in providing information on hand washing during the current COVID-19 pandemic. That is why WhatsApp has been used to share hygiene messages and prevention measures in quick and interchangeable language. Figure 1 shows the free access stickers created by users (in March 2020) with the desire to promote the prevention of COVID-19 in Spanish. These stickers, and many others in other languages, undoubtedly show a persuasive form of free access communication related to the current context of the global health emergency.

This was quickly supplemented by the WHO, which is also using stickers on WhatsApp to promote healthy behaviors ( Figure 2). By considering stickers exclusively in digital communication via WhatsApp, the WHO highlights the popularity of this non-verbal communication format and uses the full benefits of technology in favor of public health. The fact that an international entity can promote authorized and accurate information on the COVID-19 pandemic, not only allows education and promotes hygiene during the pandemic, but could also face the infodemic that affects the understanding of the current disease by civil society. ^19^ We believe that stickers are, in that sense, tools against COVID-19 and “fake news”.

On the other hand, memes are also potentially vehicles to understand the visceral manifestations of populations, as they are created by people in the exercise of their digital citizenship, they record a historical time (in which they occur) in a particular cultural way. Thus, memes are elements of a chronological record of understanding the world, in short, as an intrinsically literary element. Then, the meme can give us an account of the story of a phenomenon. ^20^^,^^21^

As the findings of this study demonstrate, nothing has yet been described about the meaning of reading a meme in health emergency contexts. We recently explained the differences between what a meme means (or what it can mean) and what it means to read a meme (what happens from the neural network to subjective mental experience when a person reads a meme) (J Moya-Salazar 2021, personal communication, 04 March). Memes become unique in communicative terms because when they are read, an exponential, inadvertent, enveloping and personal laugh comes to them. ^22^

The phenomenon of memes in the COVID-19 pandemic can also constitute a personal channel, which health institutions can use to promote health promotion activities, correct information, dissemination of hygiene measures, and disease prevention, as previously shown in other digital environments. ^13^

## Conclusions

To the date of the systematic review (December 30, 2020), no studies were identified that met the inclusion criteria related to the role of stickers and memes as tools to face the COVID-19 pandemic. These new forms of language could be important tools to inform about COVID-19 prevention strategies, since social networks via the internet are massive, fast, and interconnect population groups in real time.

The prevention strategies that governments (and civil society) are currently using are based on these “new” forms of language to undertake strategies and meet prevention indicators.

This review indicates that there are no recent studies on the subject, therefore, to estimate the role and impact of these new forms of communication in health it is necessary to develop future studies in order to improve the available scientific evidence.

## Data availability

### Underlying data

No data are associated with this article.

### Reporting guidelines

Figshare: PRISMA checklist for ‘Other ways of communicating the pandemic: memes and stickers against COVID-19’. https://doi.org/10.6084/m9.figshare.14204123.v1. ^23^

Data are available under the terms of the Creative Commons Zero “No rights reserved” data waiver (CC0 1.0 Public domain dedication).

## References

[1] Yi Y Lagniton PNP Ye S : COVID-19: what has been learned and to be learned about the novel coronavirus disease. *Int J Biol Sci.* 2020;16(10):1753–1766. 10.7150/ijbs.45134 32226295 PMC7098028

[2] World Health Organization: WHO guidelines on hand hygiene in health care. Geneva: WHO;2009.

[3] Ortiz P : Lenguaje y habla personal. El cerebro humano como sistema semiótico. In: 1ed. Fondo Editorial Universidad Nacional Mayor de San Marcos: Lima;2002.

[4] O’Reilly-Shah VN Lynde GC Jabaley CS : Is it time to start using the emoji in biomedical literature? *BMJ.* 2018;363:k5033. 10.1136/bmj.k5033

[5] Sutikno T Handayani L Stiawan D : WhatsApp, Viber and Telegram: which is the Best for Instant Messaging? *Int J Elect Comp Eng.* 2016;6(3):909–914. 10.11591/ijece.v6i3.pp909-914

[6] Carmelino AC Kogawa L : Stickers do Whatsapp: (nova) forma persuasiva de interação bem-humorada. *Rev Eletrôn Est Integ Disc Argumentação.* 2020;20(1). 10.17648/eidea-20-2589

[7] Moher D Liberati A Tetzlaff J : The PRISMA Group Preferred Reporting Items for Systematic Reviews and Meta-Analyses: The PRISMA Statement. *PLoS Med.* 2009;6(7):e1000097. 10.1371/journal.pmed.1000097 19621072 PMC2707599

[8] Munyikwa M : My COVID-19 diary. *Antropol Today.* 2020;36(3):16–19. 10.1111/1467-8322.12575 32572298 PMC7300691

[9] Gebbia V Piazza D Valerio MR : Patients with Cancer and COVID-19: A WhatsApp Messenger-Based Survey of Patients' Queries, Needs, Fears, and Actions Taken. *JCO Glob. Oncol.* 2020;6:722–729. 10.1200/GO.20.00118 32412811 PMC7271316

[10] Duong TA Velter C Rybojad M : Did Whatsapp® reveal a new cutaneous COVID-19 manifestation? *J Eur Acad Dermatol Venereol.* 2020;34(8):e348–e350. 10.1111/jdv.16534 32330322 PMC7267307

[11] Yaffe J Montgomery P Hopewell S : Empty Reviews: A Description and Consideration of Cochrane Systematic Reviews with No Included Studies. *PLoS One.* 2012;7(5):e36626. 10.1371/journal.pone.0036626 22574201 PMC3344923

[12] Munn Z Peters MDJ Stern C : Systematic review or scoping review? Guidance for authors when choosing between a systematic or scoping review approach. *BMC Medical Res Methodol.* 2018;18(1):143. 10.1186/s12874-018-0611-x 30453902 PMC6245623

[13] Becerra-Chauca N Taype-Rondan A : TikTok: A new educational tool in the fight against COVID-19? *Acta Med Peru.* 2020;37(2):249–251. 10.35663/amp.2020.372.998

[14] Martínez MM : La transmisión del lenguaje no verbal en WhatsApp. *[Thesis] España.* Universidad de Jaén: Facultad de Humanidades y Ciencias de la Educación;2019.

[15] Maffett PG : El fenómeno Whatsapp en espacios universitarios (Universidad Pública de El Alto, 2019). *Edu Sup.* 2020;7(1):46–61.

[16] Al-Maroof RS Salloum SA Hamadand MA : A Unified Model for the Use and Acceptance of Stickers in Social Media Messaging. In: Hassanien A Shaalan K Tolba M (Eds). *Proceedings of the International Conference on Advanced Intelligent Systems and Informatics 2019.* AISI 2019. Advances in Intelligent Systems and Computing;2020;1058.

[17] Carmelino AC Kogawa L : Stickers do Whatsapp: (nova) forma persuasiva de interação bem-humorada. *Rev Eletrôn Est Integ Disc Argumentação.* 2020;20(1). 10.17648/eidea-20-2589

[18] Lotfinejad N Assadi R Aelami MH : Emojis in public health and how they might be used for hand hygiene and infection prevention and control. *Ant Res Infect Cont.* 2020;9(1):27. 10.1186/s13756-020-0692-2 32041666 PMC7011445

[19] Zaracostas J : How to fight an infodemic. *Lancet.* 2020;395(10225):P676. 10.1016/S0140-6736(20)30461-X 32113495 PMC7133615

[20] Wells DD : You all made dank memes: using internet memes to promote critical thinking. *J Polit Sci Educ.* 2018;14(2):240–248. 10.1080/15512169.2017.1406363

[21] Bañuelos CJ Pérez RB : Memes e imaginarios sociales mexicanos en Copa del Mundial de la FIFA 2018. *MHCJ.* 2020;11(1):97–115. 10.21134/mhcj.v11i0.323

[22] Rodden A : Neuroscience: What makes us laugh. *Nature* .2011;473(7348):450. 10.1038/473450a

[23] Moya-Salazar J : PRISMA checklist. *figshare. Dataset.* 2021. 10.6084/m9.figshare.14204123.v1

