# Supplementary material for: Other ways of communicating the pandemic - memes and stickers against COVID-19: a systematic review
Source: F1000Res. 2021 Apr 13;10:287. [Version 1] doi: 10.12688/f1000research.51541.1 (PMC8207803; doi:10.12688/f1000research.51541.1)
Supplement: Peer review history

# Peer review: Reviewer response for version 1

**DOI:** 10.5256/f1000research.54723.r83266

**Published:** 2021-06-15

**Authors:** Jacob I. Bañuelos Capistrán

**Affiliations:** 1 Departamento de Estudios Culturales, Tecnológico de Monterrey, Monterrey, Mexico

The article explores whether there are studies related to alternative ways of communicating content related to the pandemic caused by COVID-19, such as memes or stickers, through an analysis of scientific publications related to the subject. The study finds that there are no studies on these forms of communication associated with the COVID-19 pandemic. The find is interesting. It is recommended to delve further into a detailed description and typology of these alternative forms and ignored by scientific studies on communication.

 The study has methodological rigor and provides interesting data on studies related to the use of stickers and memes to disseminate distancing and hygiene measures during the COVID-19 pandemic.

**Correction:**

 The word "Artículos" must be translated into English in Fig. 3.

 It is suggested to include a translation of the legends that accompany the stickers in Fig. 1.

Are the rationale for, and objectives of, the Systematic Review clearly stated?

Yes

Is the statistical analysis and its interpretation appropriate?

Yes

Are sufficient details of the methods and analysis provided to allow replication by others?

Yes

Are the conclusions drawn adequately supported by the results presented in the review?

Yes

Reviewer Expertise:

Image, communication, technology and culture.

I confirm that I have read this submission and believe that I have an appropriate level of expertise to confirm that it is of an acceptable scientific standard.

# Peer review: Reviewer response for version 1

**DOI:** 10.5256/f1000research.54723.r84657

**Published:** 2021-05-20

**Authors:** Kai Zhang

**Affiliations:** 1 University of California, San Diego, La Jolla, CA, USA

This is an “empty review”. The authors conducted a systematic review to determine the impact of stickers and memes as tools to cope with the COVID-19 pandemic. In order to find relevant studies, they performed a systematic search in six scientific search engines, four pre-print servers, and two general search engines. Among 8454 publications found by the automated search, none of them were considered relevant to this review study according to their inclusion criterion. Their inclusion criterion is reasonable. The fact that this review is “empty” may reflect that this area of study is very new and has not been sufficiently studied, highlighting a potential research gap in this field.

 I have no specific comments for the authors.

Are the rationale for, and objectives of, the Systematic Review clearly stated?

Yes

Is the statistical analysis and its interpretation appropriate?

Not applicable

Are sufficient details of the methods and analysis provided to allow replication by others?

Yes

Are the conclusions drawn adequately supported by the results presented in the review?

Yes

Reviewer Expertise:

Single-cell genomics

I confirm that I have read this submission and believe that I have an appropriate level of expertise to confirm that it is of an acceptable scientific standard.
